# Exploring the use of microbial enhanced oil recovery in Kazakhstan: a review

**DOI:** 10.3389/fmicb.2024.1394838

**Published:** 2024-08-01

**Authors:** Aliya Yernazarova, Ulzhan Shaimerdenova, Nuraly Akimbekov, Gulzhan Kaiyrmanova, Muhtar Shaken, Asem Izmailova

**Affiliations:** ^1^Faculty of Biology and Biotechnology, Al-Farabi Kazakh National University, Almaty, Kazakhstan; ^2^Sustainability of Ecology and Bioresources, Al-Farabi Kazakh National University, Almaty, Kazakhstan; ^3^Ecology Research Institute, Khoja Akhmet Yassawi International Kazakh-Turkish University, Turkistan, Kazakhstan; ^4^KMG Engineering LLP, Astana, Kazakhstan

**Keywords:** crude oil, oil production, tertiary oil recovery, microbial enhanced oil recovery, microorganisms

## Abstract

Microbial enhanced oil recovery (MEOR) is a promising method for improving oil recovery from challenging reservoirs such as those found in Kazakhstan. MEOR relies on the activities of microorganisms to modify the properties of the reservoir, such as reducing the oil viscosity, increasing the reservoir permeability, and generating by-products that mobilize the oil. Implementing MEOR in Kazakhstan could lead to significant economic benefits for the country by increasing oil production and royalties from fossil fuel exports. Oil production in Kazakhstan has seen fluctuations in recent years, with 2018 recording a production level of 1.814 million barrels per day. Among regions, Atyrau region contributed the most to oil production with 23.4 million tons of oil. Following Atyrau, the Mangystau region produced 8.2 million tons, and Aktobe produced 2.4 million tons. Overall, the use of MEOR in Kazakhstan’s oil fields could offer a promising solution for enhanced oil recovery, while minimizing environmental impact and cost. While specific data on the current use of MEOR in field conditions in Kazakhstan might be limited, the fact that studies are underway suggests a growing interest in applying this technology in the country’s oil fields. It is exciting to think about the potential benefits these studies could bring to Kazakhstan’s oil industry once their findings are implemented in field operations. These studies have significant implications for Kazakhstan’s oil production in the future.

## 1 Introduction

In recent years, crude oil has been the primary source of energy and a key raw material for the transport sector and chemical production. The BP Statistical Review of World Energy (BP, 2022) indicates that worldwide oil consumption rose by 5.3 million barrels per day in 2021, while global oil production increased by 1.4 million b/d in the same year. Although there is a growing interest in finding alternative energy sources, it is unlikely that conventional sources such as oil will be entirely replaced because of their significant advantages, such as high energy density and ease of use.

At the same time, oil is a non-renewable energy source. With an increase in population, there is a corresponding rise in energy consumption. Hence, it is necessary to predict production and price trends. In this situation, the use of enhanced oil recovery techniques becomes crucial. These methods aim to overcome the main obstacles to effective oil production, such as the high viscosity of crude oil, low permeability of some reservoirs, and high oil–water interfacial tension (Nazar et al., 2011; Salehi et al., 2017).

**Figure 1:**
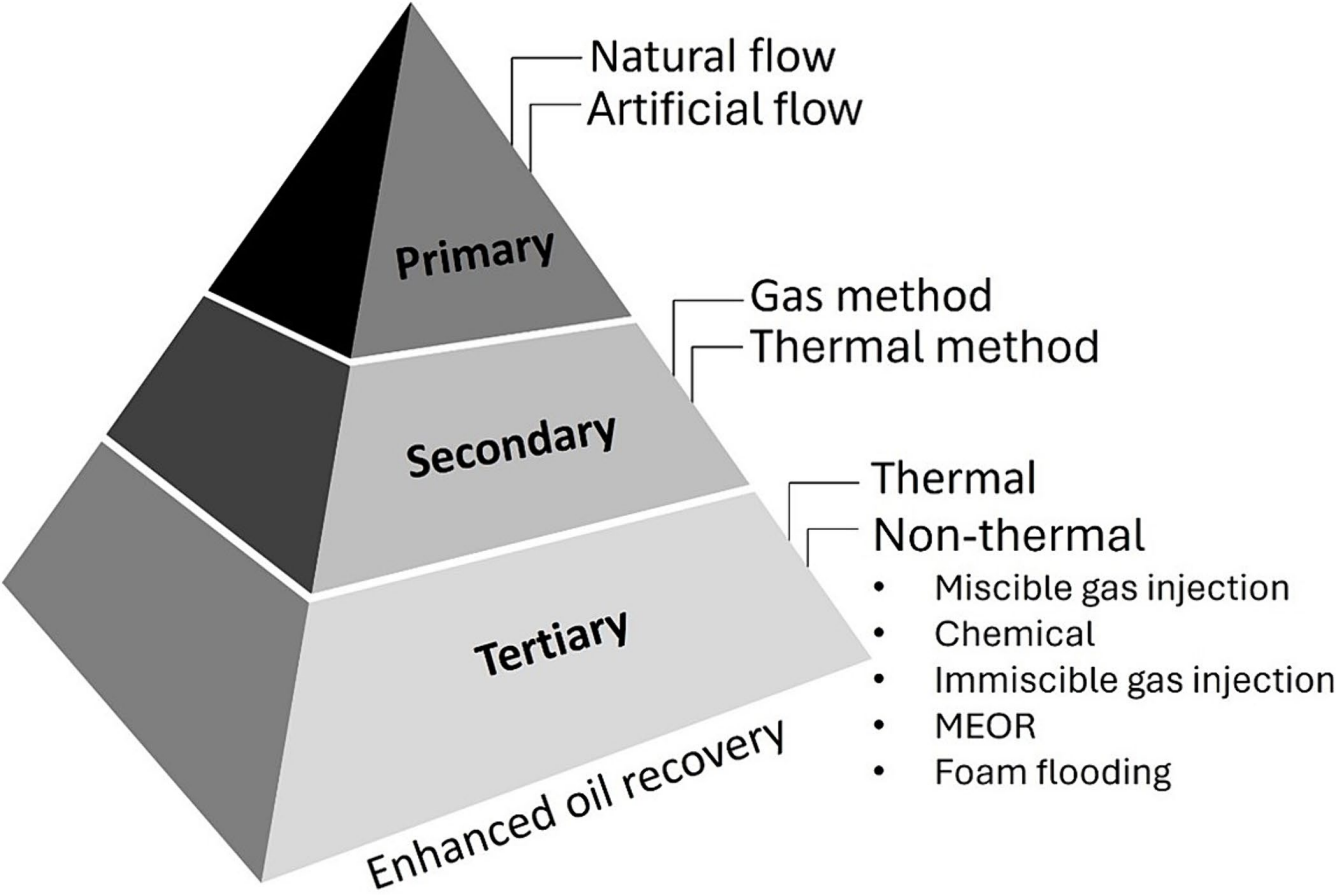
Oil production methods.

Based on the pressure in the oil reservoir and the extraction technology, there are three oil production stages: primary, secondary, and tertiary (Suflita and Mcinerney, 2008; Youssef et al., 2009). In primary production, oil is recovered using natural energy. For instance, the replacement of oil by groundwater and the expansion of gases dissolved in oil. Using the primary production, the oil recovery factor is evaluated at 5–15% (Abramova et al., 2014). When the natural pressure support is depleted, the use of secondary stage begins. External energy is supplied to the reservoir in the form of liquid (by flooding with surface or seawater) and gas (Zhao et al., 2014; Mokheimer et al., 2019). The oil recovery rate is equal to 30–40% (Deng et al., 2020). Tertiary stages of oil recovery are the ways of extracting residual reserves of oil from flooded zones during the development of hard-to-recover reserve fields. This recovery of oil production has an even better result, reaching over 40%. Alternatively, tertiary recovery is referred to as enhanced oil recovery techniques (EOR). Typically, EOR includes thermal recovery, chemical, gas injection, and microbiological methods, which can be divided into two groups: thermal and non-thermal methods (Figure 1) (Thomas, 2008; Shafiai and Gohari, 2020). Among these, the microbiological method of enhanced oil recovery (MEOR) is a promising strategy for increasing oil in old or late-stage secondary development fields. MEOR utilizes microorganisms and their metabolic by-products, which are environmentally friendly and more cost-effective (Belyaev et al., 2004; Brown, 2010; She et al., 2019). In this review, we examined the primary microbial byproducts affecting reservoir ecosystems, discussed examples of field trials worldwide, and highlighted opportunities for enhanced oil recovery in Kazakhstan’s oil fields.

## 2 MEOR and its mechanism

Microbial enhanced oil recovery can be carried out using microorganisms (*in situ*) or by injecting metabolic by-products into the reservoir (*ex situ*) (Cui et al., 2017). The microorganisms of the reservoirs are diverse and rich. They can produce a wide variety of metabolic products (Table 1). These microorganisms can influence the chemical and physical properties of reservoir rocks and crude oil (Shibulal et al., 2014). However, the growth of microorganisms depends on several factors, such as porosity, permeability, reservoir environment (temperature, pH, salinity, pressure, and nutrients), and on certain type of microorganisms (Bachmann et al., 2014). The metabolites of microorganisms have a range of advantages: biodegradability, activity in the formation environment (Lazar et al., 2007; Geetha et al., 2018; Niu et al., 2020).

**Table 1:** Microorganisms and their role in MEOR (Sen, 2008; Safdel et al., 2017; Couto et al., 2019; Niu et al., 2020; Zhang et al., 2020; Ameen, 2024; Shaikhah et al., 2024).

Microbial products	Types of microbial products	Microorganisms	Their role in MEOR
Bioacids	Propionic, butyric, acetic fatty and formic acids	*Clostridium* sp., *Bacillus* sp., and *Enterobacter* sp.	Permeability and porosity increase, emulsification, and CO_2_ produced during the dissolution of the rock.
Biogases	CH_4_, CO_2_, H_2_, and N_2_	*Clostridium* sp., *Bacillus* sp., *Brevibacterium* sp., *Methanobacterium* sp., and *Enterobacter sp*.	Increased pressure, oil swelling, viscosity reduction, permeability, and porosity increase.
Biomass		*Pseudomonas* sp., *Bacillus* sp., *Leuconostoc* sp., and *Xanthomonas sp*.	Selective plugging, wettability modification, viscosity reduction, and oil degradation.
Biopolymer	Xanthan gum, pullulan, levan, curdlan, and dextran scleroglucan	*Xanthomonas* sp., *Aureobasidium* sp., *Bacillus* sp., *Leuconostoc* sp., *Alcaligenes* sp., *Sclerotium* sp., *Pseudomonas* sp., and *Sphingomonas* sp.	Selective plugging, improve the viscosity of displacing fluid and decreasing the mobility ratio of water/oil.
Biosurfactant	Surfactin, rhamnolipid, lichenysin, emulsan, alasan, viscosin, and trehalose lipids	*Bacillus* sp., *Pseudomonas* sp., *Acinetobacter* sp., *Rhodococcus* sp., and *Desulfovibrio* sp.	Surface or interfacial tension reduction, wettability alteration, oil emulsification, pore scale displacement improvement, and viscosity reduction.
Solvents	Acetone, butanol, propan-2-diol, and ethanol	*Clostridium* sp., *Zymomonas* sp., and *Klebsiella* sp.	Rock dissolution for better permeability, oil viscosity reduction.

To carry out the MEOR process, two primary components are required: microorganisms that can either be exogenous or indigenous and can enhance oil recovery through various mechanisms, and a nutrient medium containing nitrogen and phosphorus that serves as a source of nourishment for these microorganisms (Belyaev et al., 2004; Yernazarova et al., 2016). Oil reservoir microorganisms are abundant and possess significant potential as microorganisms that can thrive under the challenging conditions prevalent in oil reservoirs. Nonetheless, it is primarily the indigenous species such as hydrocarbon-degrading bacteria, denitrifying bacteria, methane-producing bacteria, and fermentative bacteria that are beneficial for the MEOR process since they produce metabolites that aid in enhancing oil recovery (Bao et al., 2009; Safdel et al., 2017).

**Figure 2:**
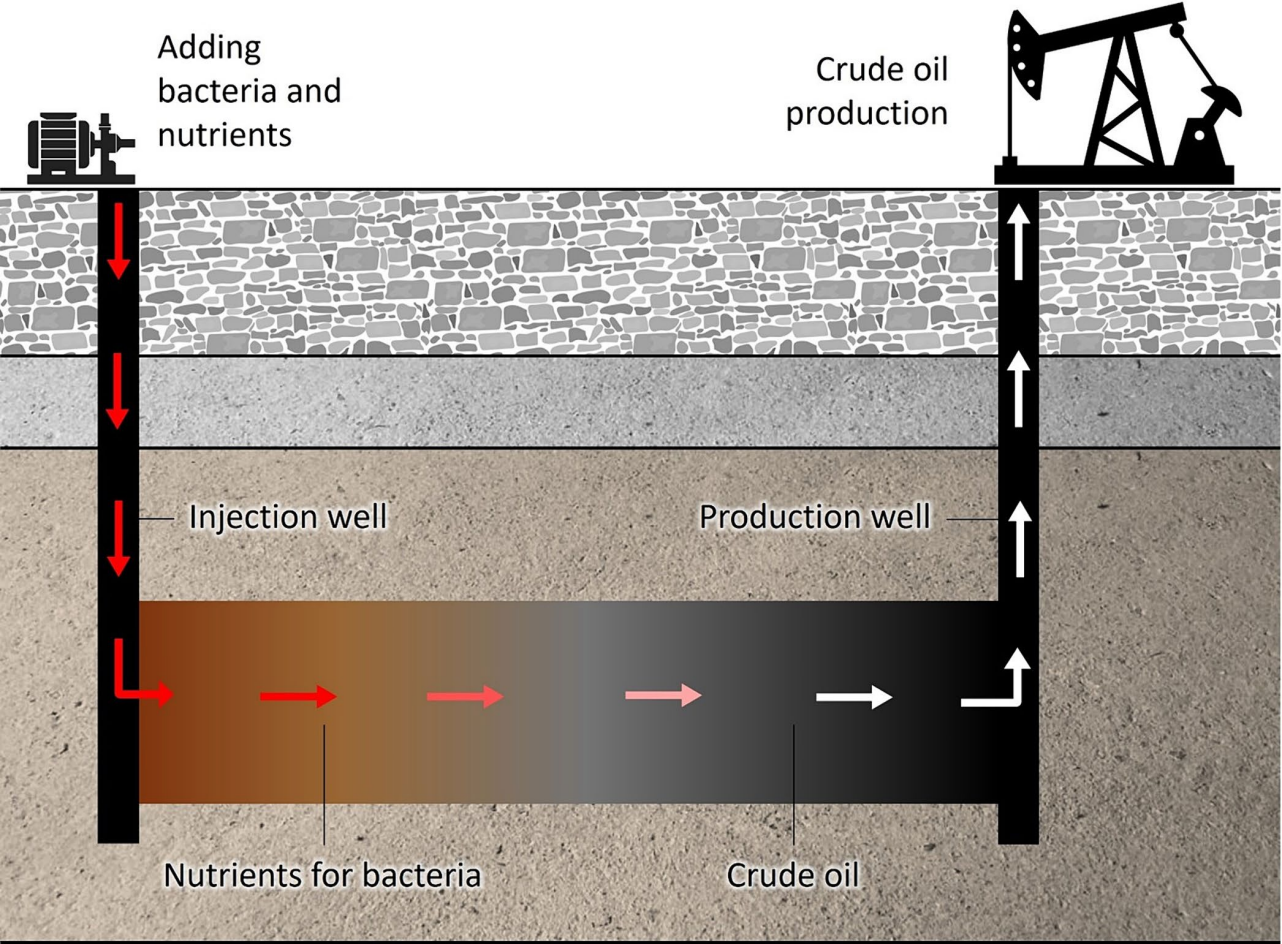
Scheme of MEOR process.

During the exogenous MEOR process, microorganisms are introduced into a mixture of water and nutrients and subsequently injected into injection well within the oil reservoir (Figure 2). The microorganisms then interact with the oil present in the reservoir by producing various bioproducts. These metabolites aid in reducing the viscosity of oil, increasing permeability and modifying the oil-rock and oil–water interfacial properties, thereby allowing for improved oil flow within the porous medium. Additionally, these microorganisms can enhance the quality of the oil by eliminating sulfur, paraffins, and heavy metals from heavy crude (Van Hamme et al., 2003; Shibulal et al., 2014).

Metabolites are the primary drivers of the MEOR process, as listed in Table 1:

- Bioacids and biogases can modify the rocks in the reservoir. This process involves the dissolution of the rock, which increases the porosity and permeability of the reservoir while decreasing the pH of the medium. Additionally, the formation of gases leads to an increase in pressure in the reservoir, causing a decrease in oil viscosity and oil swelling (Quraishi et al., 2021). Solvents play a key role in increasing oil mobility during the MEOR process. They achieve this by reducing oil viscosity. Furthermore, solvents can alter the rock wettability at the oil-rock interface, releasing oil from the porous matrix. This effect is particularly significant in carbonate reservoirs (Youssef et al., 2009). Bacteria that inhabit the reservoir are capable of fermenting carbohydrates; others release gases such as CO_2_, N_2_, H_2_, H_2_S, and CH_4_, which are then also rapidly consumed for other microbial activities. Alongside gas production, the fermentation yields organic acids like acetic and propionic acids, as well as solvents like acetone, ethanol, 1-butanol, and butanone (Sen, 2008; Niu et al., 2020; Quraishi et al., 2021). Methanogens utilize H_2_ to reduce CO_2_ to CH_4_, while sulfate-reducing bacteria transform sulfate into sulfide, and nitrate-reducing bacteria convert nitrate to nitrite and then to N_2_. Furthermore, biogases like carbon dioxide and hydrogen sulfide dissolve in water to form bioacids (Quraishi et al., 2021). Zobel introduced the concept of utilizing microbial biogases in the MEOR process, aiming to stimulate indigenous bacteria to produce CO_2_ and methane, which can restore pressure in the reservoir (Zobell Claude, 1946). *In situ* production of organic acids, specifically acetate and butyrate, by microorganisms such as *Clostridium* and *Enterobacter* can dissolve formation rocks and ultimately improve permeability (Van Hamme et al., 2003).
- A biosurfactant is an amphophilic molecule produced by microorganisms. Biosurfactants can interact with crude oil by emulsifying the oil. They can change the wettability of oil-rock interface, reduce its viscosity, and lower the interfacial tension between water and oil (Hosseininoosheri et al., 2016; Niu et al., 2020). The application of biosurfactants for MEOR has shown great potential in recovering residual oil from old oil fields (Geetha et al., 2018). Microorganisms in oil reservoirs produce a variety of biosurfactants, including lipopeptides, glycolipids, phospholipids, polymer biosurfactants, and fatty acids. *Bacillus*, *Brevibacillus*, *Streptomyces*, *Arthrobacter*, and *Pseudomonas* are known to produce lipopeptides (e.g., surfactin, lichenizin) (Dong et al., 2022). Low-molecular biosurfactants are reported to reduce surface and interfacial tension more effectively, whereas high-molecular biosurfactants exhibit excellent performance as bioemulsifiers (Geetha et al., 2018).
- Biopolymers induce selective plugging, which increases oil recovery (Jeong et al., 2019). Microbial biomass development also has the same effect, but adding biomass changes the wettability of oil formation (Zhang et al., 2020).
- Microbial biomass can be used to selectively seal reservoirs. The selectivity of the microbial plugging process is controlled by the movement of water. The proliferation of microorganisms would limit the flow of water in the high permeability region and redirect water to areas of the reservoir with higher oil saturation (Youssef et al., 2009).

There is currently a growing interest in new methods of MEOR/EOR that have been developed and explored. Various approaches have been studied and explored in this domain, including:

- Genetically engineered microbial enhanced oil recovery (GEMEOR) represents a novel approach in petroleum biotechnology. By utilizing genetic engineering methods such as recombinant technology, protoplast fusion, and mutagenesis, genetically modified microorganisms are created, combining beneficial traits from different organisms to generate more efficient strains (Sun et al., 2011; Xu and Lu, 2011; Hu et al., 2021). This approach expands the possibilities of MEOR, providing economically effective and highly efficient applications in the industry. The progress in genetic engineering opens up new opportunities for creating microorganisms capable of surviving in extreme conditions, producing significant amounts of metabolites, and utilizing readily available substrates (Niu et al., 2020). Genetic methods also allow for the development of microorganisms with improved metabolic properties, overcoming limitations faced by native microbes in MEOR (Patel et al., 2015). It is important to note that while the use of recombinant microorganisms in field applications is restricted in many countries due to environmental concerns, the focus here is on laboratory experiments. These controlled settings allow for the exploration and development of genetically engineered strains without the risk of environmental release.
- The injection of nano- and biomaterials can alter fluid flow properties, reduce surface tension, and enhance displacement efficiency, leading to increased oil recovery. For example, in a study, Khademolhosseini et al. (2015) investigated the impact of simultaneous injection of nanosilica and biosurfactant on the displacement of heavy oil in a glass micromodel. The experimental results indicate a synergistic effect of the simultaneous presence of nanoparticles and bioproducts on enhancing oil recovery. This is achieved by reducing interfacial tension, improving the mobility ratio, and increasing fluid viscosity, resulting in an approximately 58% improvement in oil recovery. Moreover, nanoparticles can serve as inhibitors to prevent asphaltene precipitation.
- Enzyme Enhanced Oil Recovery (EEOR) is a method where enzymes are employed to enhance the efficiency of oil extraction from reservoirs. In EEOR, enzymes are introduced into the reservoir to modify wettability and reduce interfacial tension. What makes enzymes particularly attractive is their capability to convert heavy components into lighter ones. Various enzymes, including proteases, hydrolases, dehydrogenases, among others, are widely applied in EEOR. Greenzyme is one of the most commonly used commercial enzyme for EOR, consisting of an enzyme and a surfactant (stabilizer) (Ott et al., 2011; Rahayyem et al., 2019; Niu et al., 2020).

## 3 Microbial community in oil fields

Subsurface oil reservoirs, previously considered inert environments, harbor diverse and dynamic microbial communities that thrive in extreme conditions characterized by highly toxic, hydrophobic, and low-activity water, as well as high temperatures and salinity. The overall microbial community structures in oil-contaminated fields are distinct and heavily influenced by environmental factors such as temperature, pressure, salinity, pH, permeability, porosity, and crude oil viscosity (Xiao et al., 2023). Despite these challenging conditions, the microbial community in oil fields plays a critical role in various processes, including oil degradation, biocorrosion, and bioremediation. Different oil reservoirs exhibit distinct microbial communities due to variations in geochemical properties and mining patterns. For instance, reservoirs with higher levels of biodegradation tend to have more diverse microbial communities, including methanogens and sulfate-reducing bacteria (Shelton et al., 2016).

Water-flooded reservoirs show significant differences in microbial communities compared to non-flooded reservoirs, highlighting the impact of water injection on microbial ecology (Wang et al., 2019). For instance, MiSeq sequencing of the 16S rRNA gene in the Xinjiang Luliang water-flooding petroleum reservoir revealed the presence of 38 bacterial phyla and 155 genera.

These are some key types of microorganisms identified in oil reservoirs:

- Thermophilic bacteria and archaea: High-temperature oil reservoirs are often populated by thermophilic microorganisms. Common thermophilic bacteria include genera such as *Thermoanaerobacter*, *Thermotoga*, and *Thermincola*. *Thermophilic* archaea include *Thermococcus* and *Methanobacterium* (Orphan et al., 2000; Lin et al., 2014).
- Methanogens: Methanogenic archaea are common in oil reservoirs, particularly those involved in methane production. They include species from the genera *Methanobacterium*, *Methanococcus*, *Methanoculleus*, *Methanomethylovorans*, *Methanosaeta*, and *Methanolobus* (Orphan et al., 2000; Grabowski et al., 2005; Gao et al., 2015).
- Sulfate-reducing bacteria (SRB): SRBs are prevalent in oil reservoirs, where they contribute to biocorrosion and souring. Common SRB genera include *Desulfotignum*, *Desulfovibrio*, *Desulfarculus*, *Desulfosarcina*, *Desulfomonile*, *Desulfscinum*, *Thermodesulforhabdus*, and others (Magot, 2005; Gao et al., 2015; Li et al., 2017).
- Halophilic bacteria: In high-salinity oil reservoirs, halophilic bacteria such as *Halomonas* and *Acinetobacter* are common. These bacteria are adapted to survive and thrive in saline conditions (Lin et al., 2014).
- Hydrocarbon-degrading bacteria: Oil reservoirs host bacteria capable of degrading hydrocarbons, such as *Pseudomonas*, *Marinobacter*, *Alcanivorax*, and *Deltaproteobacteria* (Wang et al., 2012; Sierra-Garcia et al., 2016; Davidova et al., 2019). These bacteria play a key role in the biodegradation of petroleum hydrocarbons.
- Biofilm-forming microorganisms: Microbial communities in oil reservoirs often form biofilms, which can impact oil recovery and biocorrosion. Genera such as *Thermoanaerobacterium* and *Thermococcales* are known to form biofilms in these environments (Lewin et al., 2014).
- Nitrate-reducing bacteria (NRB): NRB play a critical role in managing microbial enhanced oil recovery (MEOR) and controlling souring in oil reservoirs. NRB, such as *Sulfurospirillum* spp., compete with SRB for organic electron donors, effectively reducing hydrogen sulfide (H₂S) production and preventing souring (Hubert and Voordouw, 2007). Field studies in western Canadian oil fields have demonstrated the prevalence of planktonic NRB and their significant impact on mitigating the adverse effects of SRB (Eckford and Fedorak, 2002). Additionally, the injection of nitrate into reservoirs has been shown to stimulate NRB activity, enhancing oil recovery by increasing reservoir pressure and reducing H₂S levels (Vanhatalo et al., 2021).
- Syntrophic bacteria: These bacteria work in association with methanogens to degrade complex hydrocarbons. Genera such as *Syntrophus* and *Syntrophomonas* are involved in syntrophic associations within oil reservoirs (Siddique et al., 2011; Toth and Gieg, 2018; Sierra-Garcia et al., 2020).

Different factors, such as physiochemical and geochemical properties as well as geographical distinctions across petroleum reservoirs, combined with the introduction of microorganisms and nutrients during MEOR operations, can influence the composition of the indigenous microbial community.

## 4 Field cases worldwide

Microbial enhanced oil recovery field trials have been conducted in various countries, demonstrating the method’s potential to improve oil recovery. These trials provide valuable insights into the effectiveness and adaptability of MEOR across different geological and operational contexts. Here some notable examples.

The first MEOR field trial was conducted in 1954 in the Lisbon oil field of Arkansas, where microbes were injected along with molasses, resulting in the generation of various metabolites such as gases, acid, and biosurfactants (Anuka et al., 2020). The National Institute for Petroleum and Energy Research (NIPER) developed criteria indicating that 27% of reservoirs in major oil-producing states of the United States have potential for MEOR processes. These criteria help identify suitable reservoirs for MEOR, focusing on stripper wells where traditional recovery methods are less effective (Bryant, 1991). A successful MEOR application in Southern California involved activating indigenous microbes with a tailored nutrient blend in a mature waterflood. This treatment increased well tests from 20 to over 80 barrels of oil per day (BOPD). The process showed that managing a reservoir’s indigenous microbes could yield significant incremental oil production with minimal investment (Zahner et al., 2010). In the United States, 322 MEOR projects by 2007 showed successful results (78%) among enhanced oil recovery projects (Anuka et al., 2020).

The field tests of MEOR in China have provided valuable insights into the challenges and successes of applying this technology. These trials have extended MEOR applications to challenging conditions like high-temperature and high-salinity reservoirs, proving the method’s adaptability and effectiveness with careful microbe selection and project execution (Gao, 2018). In Daqing Oilfield, prominent results were achieved in the Chao 50 block, demonstrating a significant oil recovery increase of 3% original oil in place (OOIP) with an effective duration of 3 years (Le et al., 2015; She et al., 2019). Shengli Oilfield, as the second-largest oil producer in China, has conducted MEOR tests since 1997, with notable successes in the Luo 801 block, showing a staged actual enhanced oil recovery of 4.95% OOIP. Additionally, Changqing Oilfield conducted a pilot in an ultra-low-permeability reservoir in Ansai, indicating that microbial flooding can effectively reduce water cut and increase oil production (She et al., 2019). The Fuyu field in Northeast China implemented successful huff-puff bacterial injections from 1992 to 2001, resulting in positive responses from 195 out of 250 treated wells, yielding an additional 18,704 tonnes of oil and addressing water-cut, paraffin, and scale issues due to increased CO2 production. In the Kongdian-2 block at Dagang field, bacteria injection led to a 2–5% water-cut reduction and an estimated 17,866 tonnes of extra oil, while trials in Gangxi-3 and Gangxi-1 blocks demonstrated improved production and reduced water-cut. Xinjiang field’s bacterial treatments in 14 wells produced an additional 1,535 tonnes of oil, and nutrient injections in the 810–63 block increased output from 7.2 to 13.8 t/day. Karamay field’s 6-Zhong and 7-Zhong blocks witnessed incremental oil production of 5,399 and 21,513 tonnes, respectively, through nutrient and air injections. In the Baolige field, MEOR efforts from May 2012 increased oil production to 920 t/day, reduced water-cut to 65.8%, and achieved an additional cumulative oil production of 210,000 tonnes. Lastly, in the Chunfeng field, MEOR trials on two horizontal wells overcame water-coning issues, achieving notable breakthroughs in ultra-heavy oil production without thermal recovery methods (Gao, 2018). In Huabei Oilfield, MEOR was applied to a heavy oil reservoir with high viscosity. The method improved fluidity and enhanced displacement efficiency, leading to significant oil recovery and better injection profiles, indicating improved reservoir heterogeneity (Huang et al., 2014).

The MEOR project by Wintershall and BASF (Germany) showed promising results, with laboratory and numerical studies predicting up to 8% additional oil recovery due to mechanisms like bio-plugging, viscosity changes, wettability alteration, and reduced interfacial tension. Effective risk management strategies addressed potential issues like reservoir souring, ensuring H2S-free application, and a pilot test is planned to validate and refine these findings (Alkan et al., 2016).

Since the early 1970s, Russia has been developing microbiological technologies to enhance oil extraction. A significant milestone was the consolidation of two scientific laboratories in 1934, leading to the establishment of the Institute of Microbiology of the Russian Academy of Sciences. During the period from 1983 to 2002, numerous modifications of biotechnology were implemented at various oil fields, resulting in an additional extraction of 597,594 tons of oil in the regions of Bashkortostan (Sergievskoe oil field), Western Siberia (Bystrinskoe, Solkinskoe oil fields), and Tatarstan (Bondyuzhskoe, Romashkinskoe, Pervomaiskoe, and Novo-Elhovskoe oil fields). During this period, several MEOR methods were employed, including the basic activation of microflora near the bottom-hole zone of the injection well, activation of microflora using hydrodynamics, activation of microflora through hydrocarbon nutrition (crude oil), and activation of microflora via biological preparation. These methods were aimed at increasing oil recovery by manipulating the microbial ecosystem in oil reservoirs. The activation was stimulated by the addition of nutrients to promote microorganism proliferation in the near bottom zones, by pumping air, or by amending with H2O2 to stimulate the production of molecular oxygen, which acts as a co-factor for oxygenases (Ivanov et al., 1993; Nazina et al., 2008).

In 1986, the first pilot projects for microbial stimulation were initiated at the Magomedli Fatmai field, owned by the Binagadyneft oil and gas production department in Azerbaijan. Currently, MEOR has been successfully implemented in 19 sites within 15 fields on the Apsheron Peninsula, including Sian-Shore, Sulu-Tepe, Balakhani-Sabunchu-Ramana, Kala, Ateshgah, Kushhana, and many others. Using this method, approximately 170,000 additional tons of oil have been extracted. The effectiveness of these MEOR methods can be attributed to their focus on cultivating microorganisms in the reservoir using nutrient substrates such as molasses, whey, and activated sludge (Ibragimov et al., 2015). Suleimanov et al. (2020) introduced an innovative approach to MEOR specifically designed for oil reservoirs with highly mineralized water. This method involves injecting a low-salinity flush fluid, followed by a bioreagent composed of milk whey, resulting in a significant 74% increase in oil recovery. This approach shows promise for the development of environmentally friendly technologies and enhancing the ecological sustainability of oil reservoir management.

## 5 The petroleum sector in Kazakhstan

**Figure 3:**
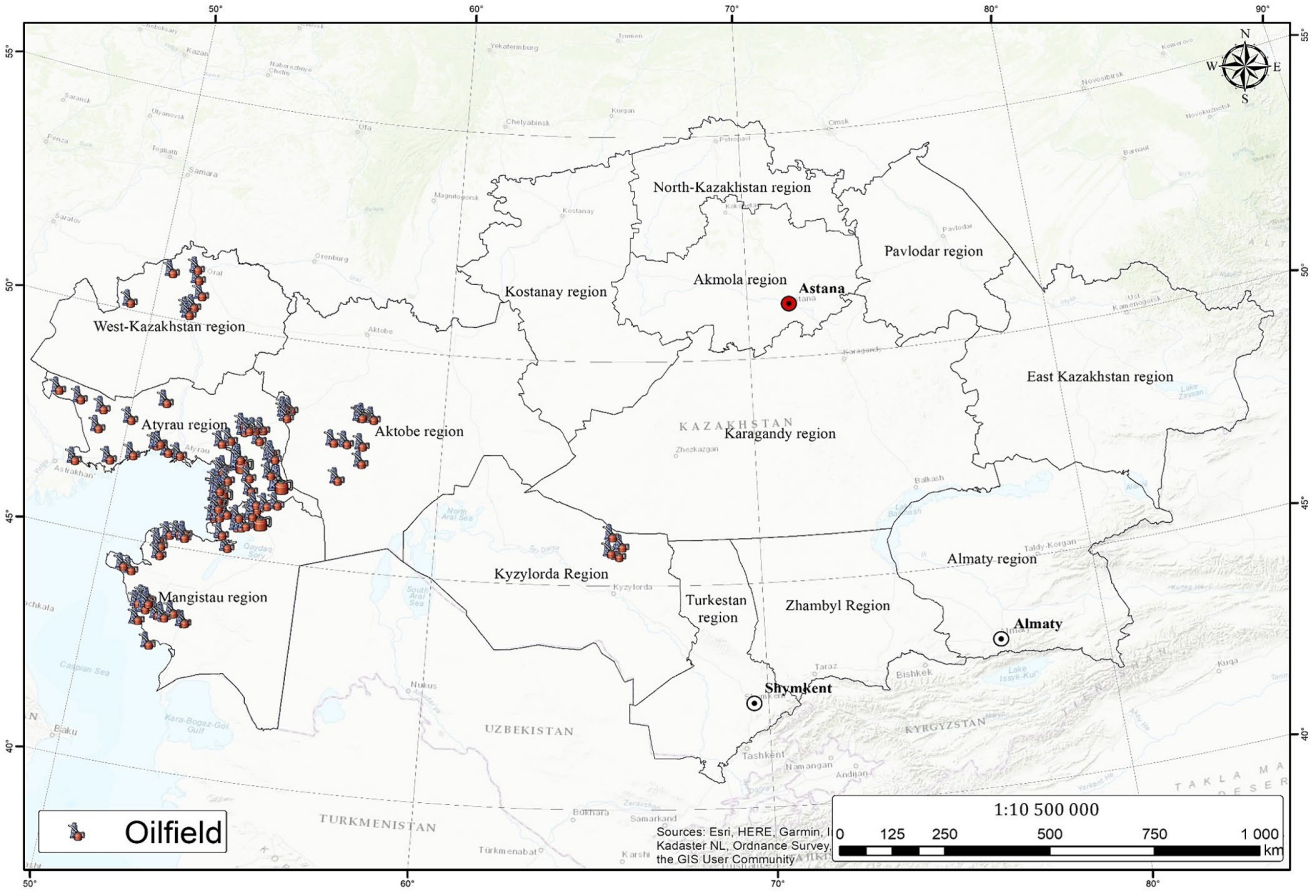
Map of the location of the oilfields. The map was created using Arcgis 10.4.

Kazakhstan is one of the world’s major oil-producing countries, with significant oil reserves and production. The oil industry in Kazakhstan has played a vital role in the country’s economy, accounting for a large portion of its gross domestic product (GDP) and exports (Kazakhstan Country Commercial Guide, 2023). The history of oil production and the functioning of the national oil market make Kazakhstan one of the traditional oil and gas-producing countries. The history of the oil industry in Kazakhstan can be traced back to the late 19th century, when the first oil well was drilled in 1899 near the city of Atyrau, much earlier than in Iran, Kuwait, Mexico, Norway, and Saudi Arabia (Khartukov, 2022). The first commercial oil well in Kazakhstan started producing oil in 1911, with a well in the Dossor oilfield yielding over 270 tons of oil in just 30 h. By the end of the 1910s, more than 350 wells had been drilled in the region, leading to a significant increase in oil production (Bealessio et al., 2021). Until recently, hydrocarbon resources were concentrated mainly in the western part of Kazakhstan (Figure 3). Western Kazakhstan can be classified into three regions based on the exploration of hydrocarbons, as identified by Blackbourn (2015):

- The Precaspian or North Caspian basin in the northwest.
- Mangyshlak, a broad peninsula bordering the Eastern coast of the Caspian.
- Ustyurt, lying between Mangyshlak and the Aral Sea.

The North Caspian basin has 45.8 billion barrels of oil equivalent, with 57% being gas. The primary hydrocarbon reservoirs are in carbonate formations, particularly in various buildups. Large deposits are present in the subsalt sequence in the Upper Devonian through Lower Permian, in structural traps and reefs. The hydrocarbon system is prolific, with high productivity in all drilled basin margins. The most productive horizons are in Jurassic and Cretaceous rocks in salt dome-shaped traps at depths less than 1,000 m. The oil has been discovered in small deposits found in pre-salt terrigenous reservoirs along the eastern edge of the basin. Most of the oils in suprasalt deposits are medium to heavy and biodegradable (Ulmishek, 2001a).

The South Mangyshlak basin contains over 40 oil and gas fields, mainly in Lower-Middle Jurassic sandstones. Sources are in Triassic deposits, and migration occurred after source maturity due to faulting and fracturing. The largest undiscovered resources are in the coastal part on the western plunge of the Mangyshlak meg anticline. Middle Jurassic sandstones in structural traps and Triassic carbonates/clastic rocks are the main reserves. Some hydrocarbons may have been generated from Jurassic clastic rocks, but reserves are likely small (Ulmishek, 2001b).

The Ustyurt basin is between the Caspian Sea and Aral Lake, bounded by late Paleozoic and Triassic fold belts. Three petroleum systems were identified with nearly 2.4 billion barrels of oil and 2.4 trillion cubic feet of gas. Oil reserves are in Buzachi Arch and Surrounding Areas of the Composite Total Petroleum System, while oil and gas fields are found in the North Ustyurt Jurassic Total Petroleum System. North Ustyurt Paleozoic Total Petroleum System is almost unexplored, but two gas flows and oil and gas shows tested in Carboniferous shelf carbonates in eastern part (Ulmishek, 2001c).

It is believed that the Republic of Kazakhstan has a considerable amount of hydrocarbon resources compared to the global reserves. The country’s proven oil reserves amount to some 30 billion barrels, making it one of the 10 biggest shareholders of the world’s hydrocarbon reserves (Orazgaliyev, 2018). As of January 1, 2019, there are more than 250 oil and gas fields in Kazakhstan. The total volume of oil reserves is about 1.8% of world oil reserves (Karatayev and Clarke, 2014). In terms of proven reserves, the Republic of Kazakhstan ranks twelfth in the world, behind the countries of the Middle East, Latin America, Russia and the United States. The Kazakh part of the Caspian Sea has a huge number of geological resources, estimated at 12–17 billion tons (Rustam, 2011). The estimated production volumes for the development of the Kazakh sector of the Caspian Sea, as outlined in the draft Decree on the State program, are: 4.356 million tons of oil, 61 million tons of gas condensate, and 518 billion cubic meters of natural gas (Decree of the President of the Republic of Kazakhstan, 2023).

In Tengiz, Kashagan, Karachaganak, Uzen, Zhetybai, Zhanazhol, Kalamkas, Kenkiyak, Karazhanbas, Kumkol, Northern Buzachi, Alibekmola, Central and Vostochnaya Prorva, Kenbai, and Korolevskoe, more than 90% of the oil reserves are concentrated in the 15 main fields (Yonggang, 2017). The fields are found in the territories of six of Kazakhstan’s 14 provinces, namely Aktobe, Atyrau, Kazakhstan West, Karaganda, Kyzylorda, and Mangistau.

**Figure 4:**
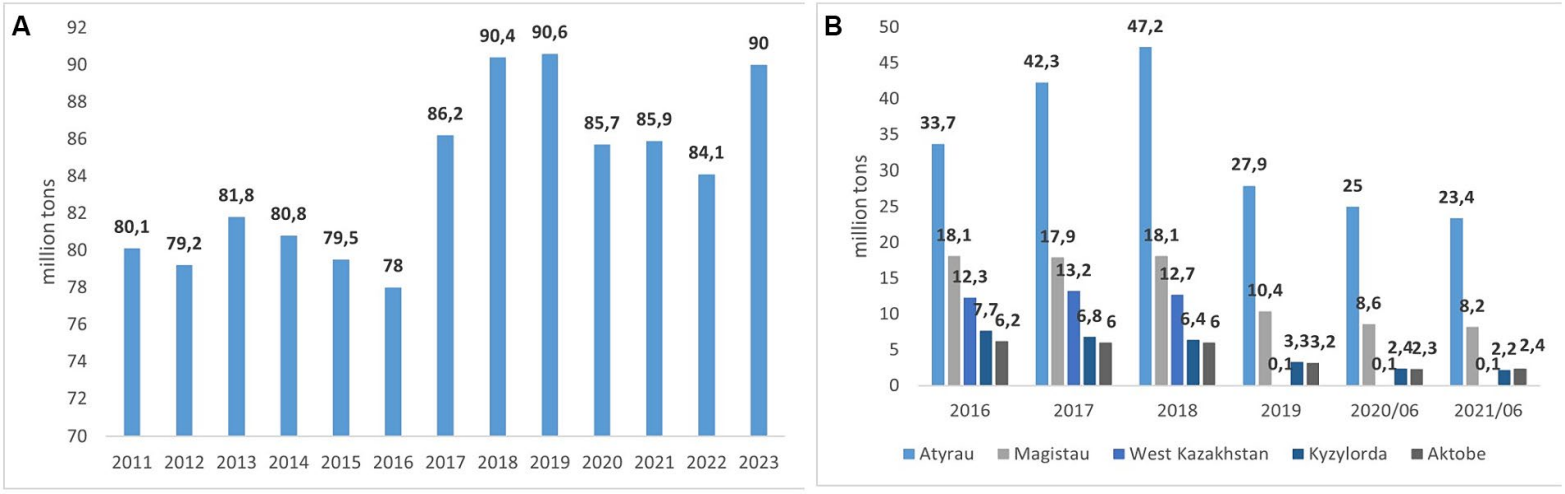
Oil production in the Republic of Kazakhstan **(A)** and by regions **(B)** (million tons).

The oil production in 2018 reached 1.814 million barrels per day. As of 2020, oil production increased by 6.84% to 1.811 million barrels per day. In 2021, oil production reached 1.8 million barrels daily (Energy Resource Guide, 2023; Kazakhstan Country Commercial Guide, 2023). According to the Committee on Statistics of the Republic of Kazakhstan, most oil production came from the Atyrau region, with 23.4 million tons of oil, a decrease of 6.6% compared to last year. The second and third places were taken by the Mangystau (8.2 million tons, down 4.2%) and Aktobe (2.4 million tons, up 0.4%) regions (Figure 4). The major crude oil and associated gas producers in the Atyrau region are the branch of “North Caspian Operating Company N.V.” (Kashagan field), LLP “Tengizchevroil” (Tengiz field), and JSC “Embamunaigas.” In the Mangystau region, the oil companies operating are JSC “Karazhanbasmunai,” JSC “Mangistaumunaygas,” and LLP “Karakudukmunay.” In the Kyzylorda region, oil production is carried out by companies such as LLP “Joint Venture Kuatam-Munai,” JSC “Turgai Petroleum,” LLP “Joint Venture Kazgermunai,” and JSC “Oil Company Kor.”

Kazakhstan exports oil through pipelines, tankers, and railways to various destinations including, Russia, Turkey, Azerbaijan, Georgia, and China. The main routes are the Tengiz-Novorossiysk pipeline to Russia’s Black Sea coast, the Kazakhstan Caspian Transportation System consisting of three segments including oil tankers across the Caspian Sea to Baku in Azerbaijan and the Baku-Tbilisi-Ceyhan pipeline to Turkey’s Mediterranean coast, and the Kazakhstan-China pipeline connecting Atyrau to Northwest China (Karatayev and Clarke, 2014; Temizel et al., 2018).

## 6 Limitations and challenges on MEOR in Kazakhstan

Kazakhstan, as a significant player in the global energy arena, actively explores various technologies with the aim of enhancing oil extraction methods. Special attention is given to the method of Microbial Enhanced Oil Recovery (MEOR) for its potential to increase the efficiency of oil extraction. In this context, it is important to consider the influence of neighboring countries, such as Russia, Azerbaijan, and China, on the development of MEOR in Kazakhstan.

Kazakhstan has seen substantial advancements in the development of oil fields, resulting in increased oil production, which has effectively contributed to the huge decrease of water use. However, these advancements have often led to challenges in oil extraction due to the unique characteristics of many Kazakhstani oil fields, which are characterized by poor permeability, high oil viscosity, and complex geological composition (Akhmetov et al., 2019).

Organizing the effective production of hard-to-recover oil deposits in Kazakhstan’s special areas, which have a great thickness of active strata, high reserve density, and are saturated with fluids that are highly paraffinic, solidifying, or viscous with high asphaltene and resin content, poses significant challenges.

In connection with the above, as oil production continues at a grandiose pace, the problem of increasing oil recovery from oil fields has become urgent. Since the 1960s, pilot projects to increase oil recovery have begun in Kazakhstan, utilizing several EOR methods, including both thermal and non-thermal techniques. Focused on hot water injection, cyclic steam injection, steam flooding, *in-situ* combustion, polymer flooding, and mixed acid gas flooding in several areas (Bealessio et al., 2021). Kudaivergenov et al. (2015) demonstrated pilot tests of gellan polymer flooding and as a result of processing five injection wells, an additional 14,585 tons of oil were produced in 19 months. Suleimanov et al. (2017) investigated the effectiveness of local acid treatment in enhancing oil recovery at the Zhetybai oilfield in Kazakhstan. They found that the treatment significantly increased area sweep efficiency, resulting in a boost in oil production. The injection capacity of wells with carbonate rocks in the formation drained zone increased by more than six times, and the area sweeping efficiency increased from 0.37 (before stimulation) to 0.46 (after stimulation).

The injection of solvents and surfactants into some reservoirs gives unsatisfactory results as the chemicals do not come into contact with the residual oil. Due to reservoir heterogeneity, injected chemicals inevitably follow paths of least resistance, where the residual oil saturation is usually lowest.

As previously stated, some exogenous and endogenous microorganisms of reservoirs can synthesize various metabolites, such as biosurfactants, acids, biopolymers, etc., that can interact with crude oil. In addition, microorganisms can help control corrosion problems in tanks (Yernazarova et al., 2016).

Interest in MEOR in Kazakhstan began in 2015. Studies on the application of MEOR methods in laboratory conditions are more common at the National Center of Biotechnology and our laboratory at Al-Farabi Kazakh National University.

The works of the National Center of Biotechnologies on microbial enhanced oil recovery began in 2015, and the metabolic activity of microorganisms isolated from the Zhanatalap and Chinarevo oil fields in Western Kazakhstan was studied (Nagmetova et al., 2015). Sarsenova et al. (2017) explored the influence of the *Bacillus Subtilis* strain J105-11, capable of synthesizing biosurfactants. A biotechnological method for enhanced oil recovery (KZ No 28836) was patented as a result.

Our team studied the diversity of microflora in the oil deposits of Western Kazakhstan (Zhetybai and Kulsary). For example, the Zhetybai and Kulsary oil reservoirs contain several genera of microorganisms, such as *Micromycetes*, *Pseudomonas*, *Bacillus*, and *Enterobacteria* (Yernazarova et al., 2016). Bacteria affiliated with the genera *Bacillus*, *Pseudomonas*, and *Desulfovibrio* were identified in the Akingen oil water under thermophilic conditions (Kaiyrmanova et al., 2018). In some projects, microorganisms capable of releasing target metabolites (acids, gases, and biosurfactants) were investigated as candidate objects for the development of a microbiological method for increasing oil recovery in depleted formations. Laboratory studies involving 15 strains of *Pseudomonas* grown on molasses bases revealed that six strains of *P. aeruginosa* (T1, T4, T5, T6, D1, and D3) exhibit significant acid-producing capabilities. The ability to produce gas was identified in eight strains of *P. aeruginosa* (T1, T3, T4, D1, D2, D6, D8, and D9). Furthermore, the maximum oil emulsification index, ranging from 50.4 to 65%, was observed in seven strains of *P. aeruginosa* (T5, D1, D2, D3, D7, D8, and D9). Due to their abilities to dilute and displace oil, five strains of *P. aeruginosa* (T1, T4, T5, D1, and D3) were identified as promising candidates for advancing microbial enhanced oil recovery in mature oil reservoirs (Kaiyrmanova et al., 2020).

Food-grade and agro-industrial byproducts are effective nutrient sources for MEOR, benefiting both ecology and economics. The most used industrial byproducts include molasses (Al-Bahry et al., 2013), corn waste (Gudiña et al., 2015), and milk whey (Yamashita et al., 2018).

Suleimanov et al. (2020) demonstrated a new microbiological method of oil recovery, with the injection of milk whey, and an oil recovery factor of 74% was obtained.

Recently, Shaimerdenova et al. (2024) explores microorganisms from oilfields in West Kazakhstan capable of producing biosurfactants and biopolymers. This study identified *Bacillus* species from the Akingen oilfield capable of producing biosurfactants, which have potential for enhancing oil recovery. The *Bacillus subtilis* A9 strain, specifically, showed strong surface tension reduction, suggesting its promise for MEOR. The study also found that salinity significantly impacts the expression of biosurfactant-encoding genes in these strains, highlighting the importance of environmental factors in MEOR applications.

Microbial enhanced oil recovery offers several benefits for Kazakhstan, including increased productivity and total oil production, as well as extending the effective life of wells and fields. Additionally, it increases the viscosity of formation water, which reduces the mobility of formation water in rocks, and is a cost-effective and low-power consumption technology compared to other enhanced oil recovery methods. MEOR is also an environmentally friendly option as microbial products are biodegradable.

The available information does not include the use of MEOR in the oil fields of Kazakhstan, as all testing is still being carried out at the laboratory level. Although MEOR is not a new technology, its development is economically viable. Resource-saving methods for enhanced oil recovery, such as MEOR, would enable Kazakhstan to increase fossil fuel production royalties and export them it to other countries.

Looking ahead, the future of MEOR in Kazakhstan holds significant promise. Continued research and development efforts could lead to the successful application of MEOR technologies directly in the country’s oil fields, moving beyond laboratory settings. Implementing MEOR on a larger scale has the potential to enhance oil recovery efficiency, thereby contributing to increased national oil production and extended well lifespan. Moreover, advancements in MEOR could position Kazakhstan as a leader in sustainable and environmentally friendly oil recovery methods, aligning with global trends toward greener energy solutions.

## 7 Conclusion

Due to the global population explosion, it is necessary to manage energy sources carefully to stimulate the world economy. At the same time, it is essential to pay attention not only to the world’s energy needs but also to the environmental consequences of the process. The ability of crude oil to meet global energy needs is undeniable, given the vast amounts of untapped reserves worldwide, including Kazakhstan. However, careful research is needed for its application in local fields, considering the geological features of the oil reservoirs and microbial diversity. The application of this method, using indigenous microorganisms, can lead to significant benefits such as cost-effectiveness, environmental attractiveness, and the development of new technologies.

## Author contributions

AY: Project administration, Supervision, Writing – review & editing. US: Conceptualization, Data curation, Investigation, Writing – original draft, Writing – review & editing. NA: Investigation, Writing – review & editing. GK: Conceptualization, Investigation, Writing – original draft. MS: Data curation, Investigation, Writing – original draft. AI: Data curation, Investigation, Writing – original draft.
